# The Easiest Becomes the Rule: Beliefs, Knowledge and Attitudes of Equine Practitioners and Enthusiasts Regarding Horse Welfare

**DOI:** 10.3390/ani14091282

**Published:** 2024-04-24

**Authors:** Letícia Santos Maurício, Denise Pereira Leme, Maria José Hötzel

**Affiliations:** Laboratory of Applied Ethology and Animal Welfare, Department of Animal Science and Rural Development, Federal University of Santa Catarina, Florianópolis 88034-001, SC, Brazil; leticiasmauricio@gmail.com (L.S.M.); or denise.nebeq@gmail.com (D.P.L.)

**Keywords:** animal behavior, management practices, social practice, sport horses

## Abstract

**Simple Summary:**

Good welfare is an emerging issue in the equestrian world, yet it is essential for those wishing to remain in the field, especially with society’s growing concerns about human activities involving animals. Recognizing this, our research aimed to understand the barriers equestrian practitioners face in implementing improvements for equine welfare. Findings indicate that while enthusiasts are aware of and acknowledge the need for good welfare practices, several factors hinder application: financial constraints, lack of physical space, insufficient skilled labor, time limitations, inadequate resources or materials, and a lack of technical knowledge. Most strikingly, despite acknowledging the need for improvement and professing love for their horses, participants did not recognize that the conditions under which they keep or use horses often fall short of their own standards. They attributed the inability to make changes not to themselves but to external circumstances beyond their control. These results pave the way for further research to determine whether equestrian activities are based on a respectful relationship with horses or if belonging to the equestrian world takes precedence, even at the expense of equine welfare.

**Abstract:**

Inadequate management conditions can impair the welfare of captive-bred horses. Understanding individuals’ viewpoints and the factors influencing their decisions about adopting or avoiding certain practices may provide insights into their motivations and decision-making processes. This is particularly relevant in the equestrian community, where equine practitioners and enthusiasts often engage in harmful practices. We explored the beliefs, knowledge, and attitudes of equine practitioners and enthusiasts about horse welfare and the barriers that prevent them from employing better management practices that are essential to promoting horses’ welfare. The study consisted of in-depth semi-structured interviews conducted in person with 31 individuals directly involved in the equestrian environment in Brazil. Responses were analyzed through thematic analysis with a data-driven deductive approach. Participants’ beliefs, knowledge and attitudes to horse welfare were divided into three themes. The first theme, “Let the horse be a horse”, captured participants’ perceptions about how physical and mental aspects related to the nature and welfare of horses. The second theme, “Everyone does it like that”, includes the social norms that influence decisions about the practices that impact on the welfare of the horses. The third theme, “Beyond utopia: how and why horses are managed the way they are”, covered barriers that participants perceived as impediments to the use of best practices for the welfare of horses. While participants demonstrated awareness of welfare issues and acknowledged factors that negatively impact horses, there was a notable discrepancy between this knowledge and the implementation of improved management practices. This could be explained by several perceived barriers to implementing management practices that could enhance horse welfare, including lack of financial resources, limited physical space, shortage of qualified labor, time constraints, inadequate tools, and insufficient knowledge. Additionally, we identified deeply rooted social norms within the equestrian community and culturally established practices that limit approaches to horse welfare. Participants underscored the influence of these norms and different interpretations of “letting the horse be a horse” based on the horse’s value and purpose. Concerning low-value horses, the primary justifications for stall housing and concentrated feeding were linked to elevated costs involved in spatial demands and labor; in contrast, for high-value horses used in performance and aesthetics, the arguments shifted to potential benefits to the horses’ well-being. From an ethical perspective, ideally, individuals should refrain from owning horses if they cannot ensure the animals’ welfare. Additionally, if the equestrian community neglects public attitudes towards animal welfare, it risks eroding its social license.

## 1. Introduction

Animal welfare refers to an individual’s state when facing environmental challenges [1] and how they subjectively perceive experiences [2]. This state includes physical and mental aspects, including the individual’s feelings [3]. Horse welfare can also be influenced by training [4] that restricts movements through the use of equipments, such as bits [5,6]. In captive-bred horses, welfare can be impaired due to inadequate management conditions [7], such as housing conditions that prevent them from socializing with other horses [8], express natural behavior [9] and forage ad libitum throughout the day, as would be the nature of the species [10]. Training can harm the welfare of horses by causing stress [4]. Similarly, inappropriate management practices and house conditions can affect horse welfare [7] triggering problems, such as stereotypic behavior [11].

The welfare state can be assessed based on physiological and behavioral measures [2], the resources available, and the quality of handling and management [7,12]. Animal welfare varies from very good to very poor [1] depending on the balance between the occurrence of positive and negative experiences [13], which is called net welfare [14,15]. But it is essential to consider that good welfare cannot always counterbalance poor welfare, as some negative experiences can be more impactful than positive ones [14]. Focusing on the promotion of positive well-being, i.e., a context of greater occurrence of positive experiences, some concepts discussed are Quality of Life [16] or “a life worth living” [17]. Specifically in the case of horses, the latter should consider the 3Fs framework (forage, friend and freedom) [18]. For instance, to live a life worth living, horses must be able to forage, socialize, and express themselves freely and subjectively perceive these experiences as positive.

Studying stakeholders’ perception plays a vital role in comprehending individuals’ viewpoints and the underlying factors that drive their choices regarding the adoption or avoidance of specific practices (e.g., pigs: [19]; cattle: [20,21,22,23]. These investigations provide valuable insights into people’s perspectives, shedding light on their motivations and decision-making processes. Equine practitioners and enthusiasts are included in different sectors of the equestrian community and seem to understand what harms the welfare of horses; however, many still employ harmful practices [24,25]. In the present study, we explored the beliefs, knowledge and attitudes of equine practitioners and enthusiasts about horse welfare and the barriers that prevent them from employing better management practices essential to promoting horses’ welfare.

## 2. Materials and Methods

This study is part of the research project “Knowledge, beliefs and attitudes of equine practitioners and enthusiasts about behaviors, emotions and welfare in horses” approved by the Ethics Committee in Research with Human Beings of the Federal University of Santa Catarina (CEPSH/UFSC), opinion n. 5,092,727.

### 2.1. Participant Recruitment

A qualitative study was conducted with 31 individuals directly involved in the equestrian environment, focusing on horse welfare. Equine practitioners and enthusiasts were considered to be those who had some form of contact with horses, such as equestrian athletes and instructors, horse owners, trainers, veterinarians, animal scientists, military personnel, university and equestrian center teaching staff, and researchers with specialized knowledge in the equestrian field. Practitioners were defined as individuals financially directly involved with horses, while enthusiasts were those with an interest in equestrian activities or indirectly involved in something that affects the horse, but not the animal directly. The study consisted of in-depth semi-structured interviews, conducted from February to May 2022, by the same interviewer in Brazilian Portuguese. The initial three participants were recruited through the authors’ network of contacts, followed by the snowball sampling method, where participants were requested to recommend additional participants for the study [26]. The initial contact with the participants was made through text messages, and in the case of agreeing to participate in the research, the interview was scheduled to be conducted in person (1 interview) or via video call (the remaining 30). Initially, 14 interviews were conducted. Subsequently, the responses were analyzed, and an additional 17 interviews were conducted. After analyzing the interviews from participants 15 to 31 in their entirety, it was found that no new themes or information were mentioned by the participants. The interviews lasted 30 min on average.

Before the interview, all participants received and signed an Informed Consent Form.

### 2.2. Interview Script

The interview started with 6 closed questions regarding socio demographic information, then participants were asked 2 open-ended questions: (1) their understanding of horse welfare (2) the perceived obstacles that prevent people from taking actions that participants believed are beneficial to horse’s welfare. The interview was categorized into sociodemographic information and knowledge, beliefs, and attitudes about welfare in horses (Appendix A). Participants were asked about their conception of horse welfare and the greatest barriers that prevent people from employing practices indicated to improve the welfare of horses. The responses were based on participants’ personal experiences with their own horses or through personal observation in other establishments. At all moments participants were given the freedom to express their opinions, beliefs, and share stories that served as examples to illustrate their opinions and experiences. The interview was structured to allow several answers. Participants could cite other types of work for horses or types of contact with horses than those described at the interview script.

### 2.3. Data Analysis

All the interviews were transcribed by the first author, who had also performed all the interviews, with participant identification using numbers to ensure anonymity. The responses were categorized into thematic topics for discussion through thematic analysis [27,28,29]. A data-driven deductive approach was employed, in which the authors were responsible for creating themes based on the raw interpretation of the data and the identification of less evident patterns through subjective analysis of factors that could be influencing the participants’ responses. To analyze these less evident patterns, a latent analysis was conducted to uncover implicit meanings beneath the participants’ superficial speech. This was performed after the initial semantic analysis, as it revealed participants’ opinions, perspectives, and beliefs that were not explicitly stated, including moral reflections about themselves and others.

The interviews were thoroughly, repeatedly, and carefully read for coding and theme development. Codes were inserted using the “Comment” feature in Google Docs throughout the document, corresponding to each quote from the transcribed interviews. Each quote was interpreted as a meaningful fragment. We sought to understand the participants’ perspectives, opinions, and experiences based on their narratives, considering the participants’ life context and searching for patterns in the data that would inform how they perceived horse welfare. Codes were created for fragments of the narratives that were deemed significant during the initial analysis or after recognizing recurring ideas among participants’ responses. Throughout the process, reflective notes and mind maps were made to capture ideas and topics related to the participants’ statements. Fragments of the narratives that were deemed significant for more than one theme were coded under different themes.

The codes were discussed among the authors to identify patterns and group the codes into themes, considering different perspectives and commonalities in the participants’ responses. Excerpts from the interviews were used to exemplify participants’ statements related to the developed themes, after analyzing the data in the co-creation of themes, avoiding paraphrasing the participants’ words. We aimed to include quotes that showcased the participants’ different opinions and viewpoints, while maintaining the original idea expressed by the participants. Finally, the qualitative and quantitative responses were organized into a table.

## 3. Results

### 3.1. Description of Participants

The study participants had diverse professional roles and extensive experience with horses. The pool of participants encompassed various roles such as handlers, veterinarians, agronomists, teachers, researchers, equine therapists, police officers, and athletes, with many participants identifying themselves as horse owners or trainers, coming from families with a long history of horse ownership (Table 1). Their involvement with horses varied, with some participants having contact with as few as two horses per week, while others were regularly engaged with up to 1000 horses per week, such as veterinary practitioners and equine reproduction. These interactions encompassed a wide range of activities including handling, sports, breeding, police work, leisure, horseback riding, equine therapy, teaching, and research.

### 3.2. Beliefs, Knowledge, and Attitudes of Equine Practitioners and Enthusiasts about Horses’ Welfare

Participants’ beliefs, knowledge and attitudes to horse welfare were divided into three themes (Table 2). The first theme, “Let the horse be a horse”, captured participants’ perceptions about how physical and mental aspects related to welfare and the nature of the equine species. The second theme, “Everyone does it like that” includes the social norms that influence decisions about the practices that promoted or were harmful to the welfare of the horses. The third theme, “Beyond utopia: how and why horses are managed the way they are”, covered barriers that participants perceived as an impediment to the use of best practices for the welfare of horses.

#### 3.2.1. Let the Horse Be a Horse

While some participants demonstrated difficulty in conceptualizing the welfare of horses (“*Ah, this question is complex! This question is very tenuous.*”—P2) others tried to explain horses’ welfare by referring to basic needs and to the respect of the nature of the species, which was captured by the expression “let the horse be a horse” (“*It’s letting the horse be a horse. As natural as possible* [as it can be]*…Let the horse be a horse. Of course, today the natural habitat of the horse doesn’t exist.*”—P21; “*Similar to their natural life, anything that is similar to their wild life is fine. For example, a muzzle is a necessary evil… On patrol we do a lot of work that goes against the horse’s nature, his nature is to run away, we make him fight.*”—P12; “*The horse being a horse,* [people] *respecting them as prey, their limits, understanding how they feel, how they think, all this is very important*.”—P24; “*A well-treated horse…a horse that has the time to be a horse… to be released in the field, that’s being a horse*.”—P6; “*…understanding it as an animal, a living being, with peculiarities, its needs… And allowing horses to have their needs met*”—P23). Participants described many aspects of the biology, physiology and behavior of the horse that they associated with a horse being well. For example, some used health as a measure to verify the state of welfare in horses (“*I see that they have good welfare if they are in good health.*”—P31; “*In addition to food, health care through vaccines.*”—P28; “*I think that* [you can say that] *a horse that is healthy, well handled,* [if they] *are going through a process where there is good welfare. They will gain weight, they will have a beautiful coat…they will eat with will, they will drink a lot of water*.”—P25), with some expressing concern about the occurrence of colics in horses associated with management (“*A stabled horse will possibly go through a colic process*”—P12; “*Colic is a proven fact, the horse in the wild has much lower chances of having a colic than that animal that is confined*.”—P30). Feeding was another frequently cited element associated with good welfare (“*The horse’s stomach is small and it fills up very quickly. That’s why we see them eating all the time…*”—P16; “*They can’t stay a long time without eating, it gives them gastritis.*”—P5). Participants also mentioned social behavior (“*…the horse is a social animal, they need to be with others.*”—P5; “*… having a friend will also generate good welfare*”.—P24; “*It’s no use releasing a horse into a round on its own, because if they don’t have the company of others, even if they can see the companion* [other horses] *on the side* [of the stall], *they don’t have good welfare.*”—P16) and the importance of freedom (“*More time enjoying freedom*”—P5; “*The freer, the happier.*”—P18) for the horses’ welfare.

Although less frequently, participants discussed the mental state as part of their understanding of horse welfare (“*Second place, mental welfare.*”—P18; “*He should not show pain, irritation… Understanding how they feel, how they think, all of this is very important.*”—P24). Some valued the opportunities for positive emotions (“*I see their happiness… they like to be with us [humans]… and they have to be very happy in what they do*”—P23) and were concerned about the stress resulting from some management practices (“*They are housed as free as possible, not in these dark stalls. You see many horses that keep biting the door, swallowing air, aerophagia, all of that is stress [example].*”—P19; “*Always bring the horse conditions that they like to be in, an environment they like. It’s no use for us to do it if they don’t like what they’re doing, right… That’s what welfare is, looking at the animal and seeing what environmental conditions they adapt to and, from that, dosing the ways to treat them according to their particularity.*”—P29). Mental welfare was also described as dependent on the people in charge of the horses (“*Management by competent, capable people who only work with positive feelings, otherwise the horse will be permanently stressed.*”—P16).

Two different views of the horse-human relationship were perceived in the participants’ conversations: an affective view and an utilitarian view. Some expressed their love for the horses, describing it in terms of friendship (“*They say that a dog is man’s best friend, but it’s the horse* [that is man’s best friend].”—P4; “*When you have a friendship between a man and a horse, it is much deeper than with a dog*.”—P21) or expressed the horse-human relationship through terms such as “passion” and “love” (“*Gauchos and southerners are passionate about horses… I am passionate about equestrian sports, I am passionate about horses, I chose to become a veterinarian because of horses.*”—P2; “*Love is what you do, it’s the important thing*.”—P20) and with sympathetic speeches (“*The cart horses… it’s a pity.*”—P4; “*I keep putting myself in their situation, if I was hungry… It’s distressing, seeing the animals hitting, asking for food, my God, what should I do?*”—P20; “*It breaks my heart, I don’t even look. Because it’s no use, I won’t be able to fight against that* [horses agitated waiting for food]*… you see their sad expression, very sad, it’s heartbreaking, so I try not to be close*.”—P24). In addition, participants referred to the communication and understanding of horses by humans (“*Perceiving the signals they give, understanding them, welcoming what they bring to us*.”—P20; “*The main secret of the horse is that you can understand what they’re telling you. They talk all the time… I think that’s the main thing, people have to listen to horses. You have to learn to think like a horse and not like a person*.”—P21).

#### 3.2.2. Everyone Does It like That

Participants’ description of how they viewed the daily practices used in horse management revealed social norms and culturally influenced knowledge. They explained their motivation, or that of others, for employing practices such as the use of stalls, individual housing, and concentrated feeding, using phrases like ‘as everyone does it’ and ‘as it has always been done (“*Some reasons, first convenience. You get out of a situation that everyone has been doing for many years*.”—P16; “*Ah, culture. Culture! … People from the countryside in general… they have the smarts of everyday life, but they have a certain ignorance and that culture is very strong*, [culture is] *ingrained in them, the traditions, how it has always been done*.”—P19; “*Some don’t do it* [adopting better management practices] *out of stubbornness, beliefs*.”—P17). This was shared even by some that believed that cultural practices seemed to be changing (“*I believe that it is more cultural, of not respecting, going beyond what the horse can go. But I haven’t seen that much anymore*.”—P23).

Some participants offered critical commentary on culturally rooted practices and challenged specific social norms (“*So, there’s this bit I’ve noticed, you know, with the ‘high-performance’ folks—they sometimes miss catching on to the importance of it, having that touch to prioritize the emotional state of the animal. Because it does have quite an impact as well*”—P24; “*But if today the horse feels that leg, they ask his veterinarian to block that pain for the test. This is a tremendous evil for the horse, but due to the greed of the human being that wants a result, they end up doing it many times*”—P30; “*The horse might not want to compete anymore, because one day it felt pain during the event and no one addressed or medicated that pain*”—P27).

Not conforming with social norms and established traditions led three participants to take radical measures like withdrawing from equestrian competitions that they considered harmful to the horses. They cited their experiences to explain why they had “given up on the system” (“*But I’ve seen horses that, when leaving the starter, rear up. It takes a lot of work, they don’t position themselves, they bend their whole body… I do horseback riding, but I don’t go to races anymore, because I started to observe this in horses*.”—P24; “*Today I’m seeing the consequences, she’s very anxious, so much so that I’ve stopped for a while, I’m not even taking her to the competition anymore*.”—P25; “*That’s why I stopped competing in that modality, because it required much more training and the pursuit of perfection is a factor that gets in the way…*”—P28).

#### 3.2.3. Beyond Utopia: How and Why Horses Are Managed the Way They Are

Participants perceived some barriers as an impediment to the use of best practices for the welfare of horses. The most commonly described management practice was keeping horses in stalls most of the time and releasing them into paddocks/pastures in small groups at specific times of the day. Participants explained that these housing conditions were associated with little socialization among the horses and a large supply of concentrated food and little forage distributed in two or three daily meals. Participants demonstrated knowledge of management practices that could improve the welfare of the horses, for example many citing that horses loose in the pasture with ad libitum feeding, as well as social contact with other horses, have better welfare. Nevertheless, there appeared to be a shared understanding that implementing such practices on a daily basis is not feasible in most situations (“*Because we can’t let them be a horse all the time*”—P7).

The primary reasons for the negative attitudes expressed towards practices that could enhance horse welfare were attributed to a lack of financial resources, physical space, qualified labor, time, tools, and knowledge. The shortage of financial resources was consistently highlighted as one of the most frequently mentioned factors (“*Sometimes people don’t have money. You see here, sometimes I have lunch, but I don’t have dinner, so I can pay the bills for the horses and dogs… If you don’t have money, you don’t have it* [horses].”—P22; “*The money factor today is what most influences* [people not doing what is essential for horses].”—P28). It was mentioned that the COVID-19 pandemic intensified the lack of financial resources (“*During the pandemic, he stayed at a farm of a friend of mine’s. I couldn’t keep two horses… We reduced work at the time of the pandemic and started to work with him later. There weren’t so many practitioners.*”—P23; “*Today, 80 students, 19 horses… Before the pandemic there were 150 students and 49 horses.*”—P29). The lack of financial resources was presented as a constraint to acquiring large areas (“*The public that consumes horses today cannot afford 1 hectare per horse… People know and don’t do it because they can’t.*”—P18; “*But having more horses is a very high cost, so it’s very difficult for you to have that*.”—P21; “*It’s difficult to have open paddocks available, where you can turn out the horses daily*”—P27).

The lack of qualified labor was mentioned as a barrier to the use of best practices (“*The caretaker is often someone earning a minimum wage, yet they’re responsible for a horse worth thousands of dollars… the challenge of workforce qualification is undoubtedly our biggest issue, along with the barrier that people face when trying to enhance their workforce qualifications… But the owner is the first to bar the qualification of labor.*”—P16; *“…labor in this area is very, very difficult.*”—P24), as well as lack of time and adequate tools (“*So, for us keepers, I think it’s sad, right. But there’s the issue of time. I’ve worked in a place where they had 30 animals, I didn’t manage to offer [the horses] everything that I believed was right. The second point that was important for me, as a horse professional, was the tools that the riding center offers me. If the hay my boss gives me is X and for him it is ok, if the concentrate is finished and he says, solve it, my hands are tied.*”—P20; “*Some say, my management doesn’t allow it. Because of the routine, they can only feed the horses 2 times a day, they cannot insert a third [meal] unless it is at midnight.*”—P16).

It was clear that the welfare of horses is often associated with their usefulness and economic value (“*Running a horse facility, no matter how basic [the facility is], is a lot of work and needs to be a source of livelihood. You have to take all of that into consideration. Not meeting the needs of the horse involves so many things. …A sport horse is well taken care of within what’s feasible, no one is going to mistreat a $70,000 horse, they’ll take good care of such a horse. People in equine therapy aren’t going to mistreat their primary therapist. However, there are still many carters out there with that perspective that the horse is there to serve you, you know?*”—P23; *“…a sport horse, a horse that people pay the price of a car for, expecting a financial return, it’s an animal that will yield results, right… people take great care of it.*”—P2). Physical and social isolation seemed to be justified by the value and use of the horse (“*There are horses that are super delicate to be released in the paddock, because they really hurt themselves, a lot of energy, a lot of euphoria, stallions mainly, right? They really end up being deprived of a lot of things, because they hurt themselves or hurt other horses or people.*”—P27; “*You can’t let the younger ones [horses] go all together, they fight a lot… There’s not much to do. If you let them go, it’s worse, because they fight, they get hurt easier.*”—P13). These comments were made mainly to discuss the case of sport horses (“*These horses [referring to sport horses] are animals that often eat a lot… so sometimes they end up not having some privileges, because they get hurt.*”—P26) and those that needed to achieve an expected performance (“*Athlete horses, like humans, go through stress to be able to maintain weight and avoid wearing out the musculature at the wrong time. So a horse that is prepared for sport or for handling is a horse that will have to be confined.*”—P2; “*My horses are loose in the pasture at some periods of the day and kept in stalls at others, because they are sport animals, they need to be prepared for sport, to compete.*”—P5). A third justification for isolating horses in stalls was the aesthetic value of the horses that participated in competitions or exhibitions (“*How are you going to have a stallion that goes to exhibitions to be released in the field? There’s no way. He’ll be ugly, he can throw himself on a fence.*”—P5; “*We bring them to the stables to leave them with the most beautiful coat, shoed hoof, leave them with the conditions to be presented. A beautiful horse.*”—P7; “*But when it comes to competition, then you think about the morphology or the functional part. Whether you like it or not, you deprive them of what would be ideal welfare.*”—P25; “*Nowadays it’s very competitive, people start to stable the foals when they are born… If the foal is born very beautiful, they begin to participate in a category called “incentive”, which is to encourage horse keepers.*”—P6). Some recognized the trade-off between performance and welfare that justified their support for isolating horses (“*Sometimes it’s difficult to fully respect their welfare, let’s put it that way, I think it’s a necessary evil for anyone who wants to compete.*”—P26; “*Some horses we need to release separately, because they fight, there are troublemakers. But this separation is harmful for the animal, because they have this collective need.*”—P9; “*Because when you ride, or in a certain way use a snaffle…I believe that it is not good welfare, but this is the reason why we keep the horses*”—P19).

## 4. Discussion

Among the different points of view of the participants, many equine practitioners and enthusiasts’ perceptions about horse welfare were in line with aspects related to the welfare of horses discussed in the scientific literature, with a specific focus on horses’ basic needs and adherence to the 3Fs Framework—freedom, forage, and friends [18]. Elements perceived by participants as integral to ensuring a high state of welfare encompassed group rearing on pasture, distributing meals throughout the day, providing access to hay and water ad libitum, and implementing strategies to foster positive experiences while mitigating negative ones like stress and pain, particularly in sport horses. However, our analysis revealed a disconnection between what participants deemed essential for good welfare and the management practices they employed or endorsed. This was associated with several perceived barriers to implementing management practices that could enhance horse welfare, including lack of financial resources, limited physical space, shortage of qualified labor, time constraints, inadequate tools, and insufficient knowledge. Deeply entrenched social norms within the equestrian community and culturally established practices played an important role in hindering changing approaches to horse welfare. Participants underscored the influence of these norms and different interpretations of “letting the horse be a horse” based on the horse’s value and purpose. Concerning low-value horses, the primary justifications for practices such as stall housing and concentrated feeding were linked to elevated costs involved in spatial demands and labor. In contrast, for high-value horses used in performance and aesthetics, the arguments for keeping horses in stalls shifted to potential benefits to the horses’ well-being. We suggest that participants cited potential benefits to horse welfare, such as performance, but qualified labor and physical space could also be considered.

When discussing their conception of horse welfare, participants referenced various ethical considerations addressed by prominent scholars. These included the nature of the species (*telos*) [30], considerations of horses as sentient beings and their health [31], as well as human-horse interactions [2] and attention to basic needs [32]. Other studies also showed that people implicated in the use of animals, including horses, are concerned about natural life [33,34], physical space and freedom [33,35], health [34,36], feeding, and human–animal relationship [36], affective states [34,37], with the inclusion of pleasant experiences [38] as valuable elements of the welfare concept. The focus on the affective states of animals is in line with the consideration of the mental aspect of the animals in the assessment of animal welfare [2] and the fact that horse practitioners attribute high sentience capacity to horses [39]. However, concerns for negative emotions often did not extend to health issues, such as recognizing pain. People struggle to identify the cause of pain in horses [40], which is often a subtle sign of diseases like gastritis in horses kept in stalls. Participants also highlighted the quality of the human–horse relationship as integral to animal welfare. It is well-recognized that handlers’ work and their relationship with animals significantly impact animal welfare [41]. This relationship is formed based on successive positive and negative experiences [42], so people responsible for the care of horses must focus on maximizing the experiences that horses perceive as positive.

Our findings highlight a dissonance between the practices and the views/knowledge of equestrians, a phenomenon previously noted by others [25,43]. Participants endorsed practices that do not allow freedom to graze, express natural behaviors, and socialize with peers, such as maintaining horses most part of the daily time at stalls, contradicting their conception of animal welfare. The generalized use of stall housing, social isolation and concentrated feeding reported in this study has been documented by others [8,25]. Participants’ justifications for these practices resonate with previous research in different global contexts, including financial limitations [25,44,45,46,47,48], lack of physical space, labor [25,43], time [47], and lack of knowledge [35,44,46,48].

Inconsistencies between participants’ conceptualization of equine welfare and the actual practices they implemented could potentially give rise to psychological discomfort, as evidenced by the contradictions in their statements. Such inconsistency between beliefs and behaviors may be explained by the theory of cognitive dissonance [49]. Participants accommodated their dissonant attitudes and opinions with arguments lacking technical or scientific basis. For instance, they rationalized the practice of keeping horses in social isolation based on the erroneous belief that the more excitable personality of certain horses could lead to fights. Similar discomfort was also noticed among participants in another study when they overlooked the association between “behavioral problems” and horse welfare, attributing such problems to individual characteristics of the animal [43]. We propose that the barriers to adopting best practices cited in our study are not inherently insurmountable but are perceived as such [50]. Participants had choices, such as changing some practices or ceasing breeding horses—as reported by some participants who, at some point, decided to align their actions with their moral values. Other participants improved housing conditions to promote the welfare of horses, exemplified by modifications to stall architecture that enabled horses to see their neighbors, a practice shown to improve horse welfare [51].

Although some participants expressed sympathy or pity for the horses kept under suboptimal conditions, they did not appear to experience the negative emotions of the horses in a sensory way that could imply empathy [52], nor did they implement efforts to alleviate the suffering of the animals. Similar to a study involving pig farmers [19], sympathetic attitudes and recognition of the animals as sentient individuals were frequent but not enough for participants to support or adopt practices that meet the behavioral needs of horses. Expressing love for horses without corresponding adherence to best practices was not unique to this study [8,43,48]. Our findings suggest that this dissonance does not reflect a lack of empathy or sympathy for horses but rather a distinct understanding of what is best for a horse according to its purpose.

According to Wilkins et al. [53], people tend to inconsistently attribute emotions to non-human animals, with such attributions largely influenced by the perceived utility and cognitive status of the animal and its position within a human-centric hierarchy scale. This conflation of an animal’s being with its utility or value suggests a natural inclination among humans to blur the lines between affection directed at a horse and affection for activities involving the horse. This notion is further complicated by the fact that equestrian activities typically require the support of a well-defined peer group to which people tend to attribute a significant social value, as well as to the act of participating in these groups. Research on the role of peer groups in human behavior and mental health, such as the work by Laursen and Veenstra [54], underscores the critical role of social belonging in human well-being. Ultimately, the question arises whether the affection people have is genuinely for horses or the sense of community found within some part of the equestrian community. In this context, enhancing the welfare of horses without noticeable results for practitioners may be the reason for the low motivation of some equestrian enthusiasts to promote genuine horse welfare.

The perpetuation of many of these practices was associated with their widespread acceptance and preservation within the equestrian community of the participants. Traditions, social norms and culture can hinder changes that promote animal welfare [20,44]. Social norms are among the justifications for low levels of equine welfare [44]. The participants in our study had long experience with horses, as reported by other authors [37]. This may explain why this culture was so strong and why practitioners followed the practices. Due to their widespread adoption within the equestrian community, participants may overlook the problematic nature of certain practices that harm horse welfare. Belonging to a group influences people’s worldview [55] and being part of a group that considers these practices acceptable can make people feel comfortable, moving away from the problem and shifting responsibility to other group members. The Theory of Social Practice [56] explains that everyday practices, called “social practices”, represent a social system’s values and beliefs that people can preserve over time or transform. Social practices result from three interrelated factors [56]: firstly, materials, here identified in the financial resources, space, skilled labor, time and tools available to care for the horses; secondly, meanings, i.e., the culture of using the horse; and, thirdly, skills, represented by the participants’ knowledge about horse welfare and the practices that can be utilized to manage horses. The result of the interconnection among these three elements can explain why some practices have been preserved over the years in the equestrian environment, configuring barriers that prevent the transformation of values into action [56]. However, traditions or convenience are not morally acceptable reasons for implementing practices that harm animals [57].

The discrepancy between the notion that horse welfare is connected to letting the ‘horse be a horse’ and the practices outlined by participants was especially noticeable for horses in equestrian competitions. This highlighted a distinction between economically less valued horses and horses of higher economic value, commonly labeled ‘sport horses’ by participants, as a distinct category within the equine population. For horses of lower economic value, the notion of ‘letting the horse be a horse’ was characterized as ‘utopian’, given the limited resources available for their care. However, for sport horses, the expression implied different practices aimed at optimizing the horse’s performance, even if it meant compromising their welfare. This horse category was perceived to possess specific requirements tailored to achieve particular objectives, such as winning competitions. This perception justified participants in implementing measures, such as social isolation and concentrated feeding, to address the specific needs of the sport horse. Remarkably, when discussing the poor management of these horses, they often attributed the behaviour to ‘others’ while citing personal experiences and assuming responsibility for practices that limit the freedom and natural behavior of high-value horses or involve inadequate training schedules. Luke et al. [43] also found a difference in concern for high-value and lower-value horses among amateur equestrians. Leme et al. [8] reported that high valued horses were more often kept in stalls than low value horses. In another study [58], the difference in the perception of welfare was not related to the value of the horses but to levels of equestrian sport, with the horses used in competitions considered at a “higher” level (considered sport horses) having welfare perceived as better than “inferior” competition horses (considered just horses), except for psychological health. Although participants in our study and the study by Furtado et al. [58] referred to different factors (value of the horse and levels of equestrian sport, respectively), the conclusions of the two studies were similar.

The progression of societal practices, mainly those potentially detrimental to the welfare of sport horses, can be influenced by public pressures. For instance, practices like nerve blocking for alleviating horse pain, frequently cited by participants, may be linked to media-driven awareness of welfare issues in these animals [48]. The media’s ability to amplify public visibility regarding horse treatment during competitions [58] holds sway over public opinion on animal-related issues [59] as well as consumer purchasing decisions [60]. Specific issues may only come to public attention when visibly evident during competitive performances, whereas the routine care of sport horses when not in competition is often overlooked, forgotten, or inaccessible to the public [58]. The public has a growing influence on the decisions and practices employed by large organizations [61,62,63]. Today, there is much discussion about the social license to operate, a concept that explains an unofficial contract or license whereby the public attributes legitimacy to the operation of an organization or activity [64,65]. The collective force of public awareness, media coverage, and public pressure can drive changes, including establishing regulatory norms to safeguard animals. In Brazil, consumer concerns about farm animal welfare and preferences for more natural animal production systems have triggered changes [66]. However, horses, unlike cattle, are not generally considered “food” in most parts of the world, potentially rendering the equine industry more vulnerable to public scrutiny, as their use by humans may seem less justifiable. Individuals within the equine industry are apprehensive about potential social sanctions [43]. They must consider public ethical concerns [57] and recognize their reputation as a motivation to improve animal welfare patterns [44].

The methodology chosen for selecting research participants was the “Snowball technique” method, through which one interviewee nominated others to participate in the research. In Brazil, equine practitioners and enthusiasts are mostly men, and this trend was reflected in our study. Since the research was qualitative, we cannot determine if or how the gender distribution may have influenced the results. Considering the qualitative nature of the research, the obtained results reflect the beliefs, knowledge, and attitudes of this specific sample of interviewees. To minimize biases introduced by sociodemographic and factors related to the participant, we included participants involved with horses in various capacities, such as handlers, veterinarians, agronomists, teachers, researchers, equine therapists, police officers, and athletes.

We suggest that the discussion about the impact of management practices on horse welfare encompasses the reasons why people do not implement better practices, such as financial limitations, lack of labor, social norms, cited in our work. Knowing those barriers and related factors may be useful to convince equine practitioners and enthusiasts to challenge common sense, improve horse welfare and change human-horse relationship.

## 5. Conclusions

This study showed the beliefs, knowledge and attitudes of equestrian practitioners and enthusiasts about the conception of the welfare of horses, perceived barriers to promoting better practices for horses’ welfare and the social norms of the group that influence their practices. Participants oriented their practices according to the horse’s economic value or purpose, and many expressed no intention of changing them or challenging the culture and status quo of the equine industry. Although participants showed an understanding of horse welfare and the factors that negatively affect it, this knowledge did not translate into the adoption of practices aimed at improving welfare. The justification for why they and others did not intend to change practices that could enhance horse welfare was often anchored on social norms in the equestrian world. The sense of belonging within the equestrian community seemingly holds more significant value than the ethical principles and commitment to ensuring the welfare of horses.

## Figures and Tables

**Table 1:** Demographics of participants.

Demography	*n*	%
Sex		
Female	9	29%
Male	22	71%
Time of experience with horses		
Until 5 years	3	10%
6 to 10 years	3	10%
11 to 15 years	3	10%
16 to 20 years	3	10%
More than 20 years	7	23%
Since childhood	12	39%
Level of education		
Elementary school	1	3%
High school	7	24%
University education	21	70%

**Table 2:** Themes and subthemes that explained participants’ beliefs, knowledge and attitudes to horse welfare.

Theme	Subthemes
Let the horse be a horse	Basic needs and respect the nature of the species
	Mental state
	Different views of the horse-human relationship
Everyone does it like that	Social norms and culturally influenced knowledge
	Critical commentaries/challenged specific social norms
	Radical measures like withdrawing from equestrian competitions
Beyond utopia: how and why horses are managed the way they are	Lack of financial resources
	Lack of physical space
	Lack of qualified labor
	Lack of time
	Lack of tools
	Lack of knowledge
	Horse usefulness and economic value

## Data Availability

Data are unavailable due to privacy.

## Appendix A. Script Interview about Horse Welfare

- Demographic information:
  (1) Level of education
    Elementary school
    University education
    Post graduate
  (2) Sex
    Female
    Male
  (3) Time of experience with horses
    Between 1 and 5 years
    Between 6 and 10 years
    Between 11 and 20 years
    More than 20 years
  (4) Type of contact with the horse
    Owner
    Handler
    Veterinarian
    Zootechnic
    Student
    Researcher
    Equestrian athlete
    Trainer
  (5) Number of horses you have contact with
    Between 1 and 5 horses
    Between 6 and 10 horses
    Between 11 and 20 horses
    More than 20 horses
  (6) Type of work your horses are employed in
    Horsemanship
    Researcher
    Mounted police
    Tour
    Rural/adventure tourism
- Knowledge, beliefs and attitudes about anticipatory behavior in horses:
  (1) What do you understand about horse welfare?
  (2) What obstacles prevent people from taking actions that they believe are beneficial to horse’s welfare?
